# Technique for secondary modification after maxillary resection and reconstruction for soft tissue flap fixation before prosthesis addition: a case report

**DOI:** 10.1186/s12903-019-0821-6

**Published:** 2019-06-21

**Authors:** Atsushi Abe, Kenichi Kurita, Hiroki Hayashi, Yu Ito

**Affiliations:** 1 Department of Oral and Maxillofacial Surgery, Nagoya Ekisai Hospital, 4-66 Syounen-cho Nakagawa-ku, Nagoya, 454-8502 Japan; 2 Department of Oral and Maxillofacial Surgery, Aichi Gakuin University, Nagoya, Japan

**Keywords:** Secondary modification technique, Reconstruction, Flap fixation, Maxillary cancer, Vestibuloplasty, Prosthesis, Case report

## Abstract

**Background:**

The removal of maxillary carcinoma causes various types of tissue defects, which can be corrected by free flap reconstruction. In flap reconstruction after maxillary cancer resection, ensuring prosthesis stability is frequently difficult owing to the flap’s weight. Therefore, a second modification technique is required for improvement of configuration. This case where flap suspension and flap modifying surgery were performed using anchor system for the extensive complete maxillectomy case.

**Case presentation:**

The patient was a 56-year-old male, who underwent an extensive total maxillectomy and flap reconstruction using the rectus abdominus muscles in May 2005. Postoperatively, due to the difficulties of wearing a maxillary denture, he was transferred to our department with the chief complaint of morphological improvement. The maxillary bone had already been removed from the midline with the rectus abdominus muscle flap sutured directly to the soft palate without oral vestibule, and the flap margin was moving together with the surrounding soft tissue. The flap size was 70 × 50 mm, which was sagging due to its own weight and was in contact with mandibular molars, reducing the volume of the oral cavity without a denture being worn. Flap reduction and lifting the flap were performed under general anesthesia using 3 Mitek anchors implanted in the zygomatic bone, and the anchor suture was placed through the subcutaneous tissue to lift the flap. Postoperatively, the prosthesis was stable. No recurrence of flap sagging or wound infection was seen 3 years after surgery.

**Conclusions:**

The second modification technique after maxillary cancer resection is useful for ensuring prosthesis stability. This method can be used before prosthesis addition. We could obtain remarkable denture stability by flap suspension using anchor system and a flap-modifying operation for the patient who had undergone maxilloecotomy. The denture was stabilized by using anchors for the elevated flap and flap loss technique and by performing vestibuloplasty for support.

## Background

Treatment of massive malignant tumors of the maxilla can open communication between the oral, nasal, and orbital cavities, thereby resulting in hypernasal speech, food, and liquid countercurrent in the nasal cavity, dysphagia, masticatory disturbance, and facial disfigurement [1–4]. Free flap reconstruction can be used to repair various tissue defects resulting from removal of a maxillary carcinoma [5]. The sagging of a thicker flap can result in insufficient denture space or cause the denture to fall out, requiring a secondary modification surgery similar to pre-prosthodontic surgery to improve and expand the alveolar ridge for better denture support [6].

To obtain denture stability, it is necessary to eliminate the factors that raise the denture border by cutting the muscle origin and the frenulum as well as expanding the area for the denture base. However, this method has limitations in morphological improvements and cannot prevent flap sagging in cases in which rigid reconstruction of the rectus abdominis muscle flap was not performed. Several studies have reported the effective use of anchors to prevent the sagging of a bulky flap [7–11] .

This clinical report describes a secondary modification technique for use following maxillary reconstruction and reconstruction of soft tissue flap fixation prior to adding a prosthetic device.

## Case presentation

**Fig. 1:**
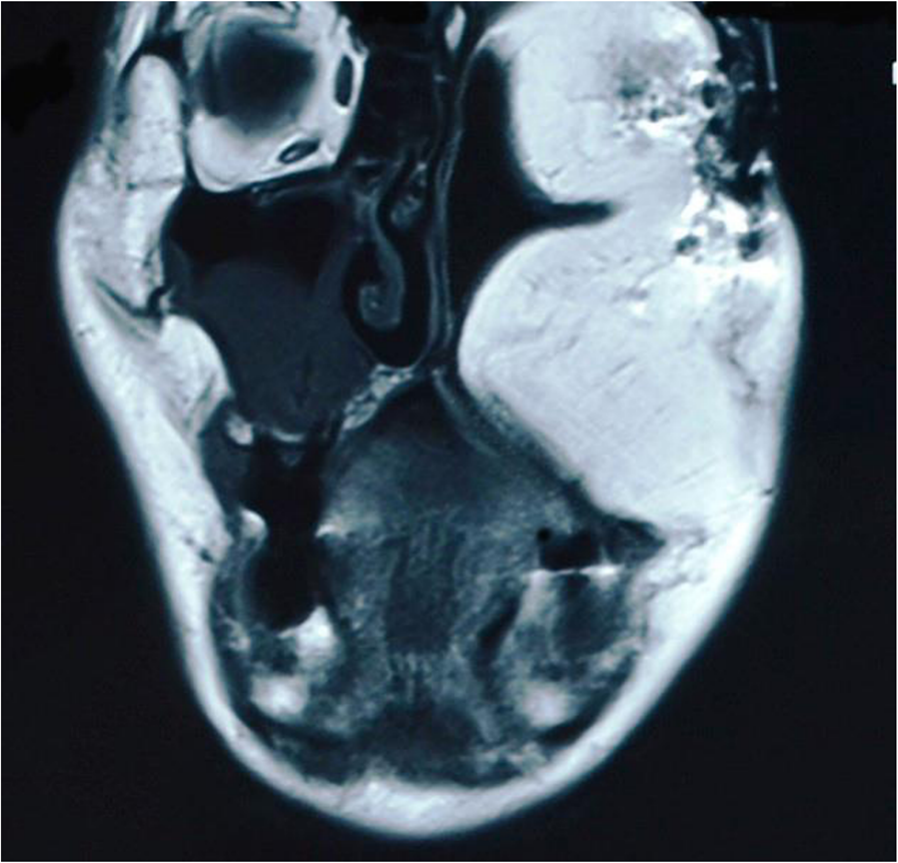
No dead space was observed due to placement of abdominal rectus muscle from anterior cranial base to oral cavity during reconstruction

In June 2011, a 56-year-old male was referred to our department by head and neck surgeon in order to improve his upper denture retention and stability. The patient was diagnosed with a squamous cell carcinoma of the maxillary gingiva (T4N0M0) in May 2005 and underwent an extended left maxillectomy, an anterior and middle cranial base resection, a left ophthalmectomy, and a flap reconstruction using the rectus abdominis muscle were performed. On physical examination, a recessed deformation on the left side of his face could be seen because of the left ophthalmectomy. The function of the left levator palpebrae muscle was eliminated to the level of a slight elevation by using the frontal muscle. A metal plate was anchored to the inferior wall of orbit. The left ethmoid bone, inferior nasal turbinate, the maxilla, alisphenoid, medial and lateral pterygoid muscle were already excised during the mesh titanium plate reconstruction of the anterior wall from the maxillary orbital region. Intraorally, the left maxilla had been excised from the midline, with the rectus abdominis muscle flap sutured directly to the soft palate. The peripheral mucous membrane around the left upper lip was already scarred, without the oral vestibule, and the flap margin had moved along with the surrounding soft tissue. The 70 × 50 mm flap was sagging from its weight and was in contact with the mandibular molars, reducing the volume of the oral cavity unless dentures were worn. The maxilla was removed from the midline to the maxillary tuberosity, while the mandible was removed from the anterior border of the ramus to the coronoid process. Dead space was eliminated because the abdominal rectus muscle was placed from the anterior cranial base to the oral cavity during reconstruction (Fig. 1). No expiratory leakage or food reflux was observed, and the rhinopharyngeal closure was maintained. Prior to performing surgery, there was no tumor recurrence or metastasis. The patient had a mouth opening of 43 mm, which we judged operable and then conducted the flap reduction and elevation under general anesthesia in Dec 2008. Informed consent was obtained from the patient’s parents prior to study initiation, and all procedures were performed in accordance with the Declaration of Helsinki.

**Fig. 2:**
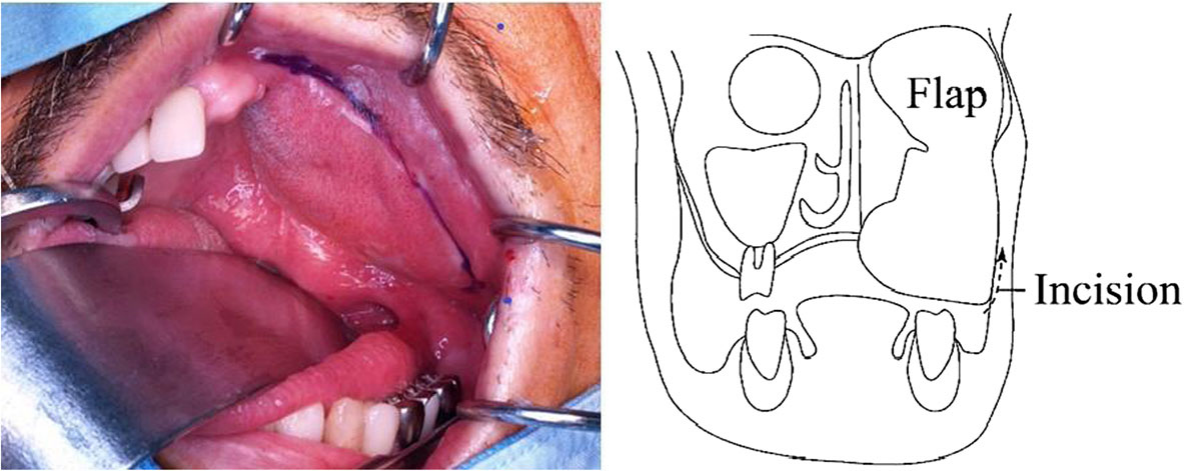
Incision was made from buccal side of sutured edge in abdominal rectus muscle. Adipose tissue was peeled from buccal side to slightly beyond skin flap center while maintaining approximately 5-mm thickness

**Fig. 3:**
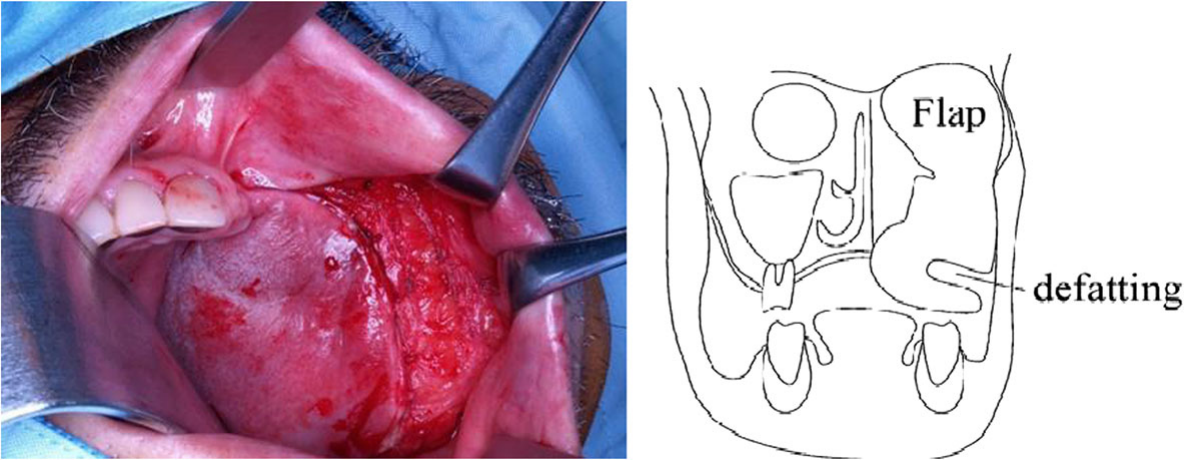
Using radio knife (8 g), adipose tissue was removed from buccal side to point slightly exceeding skin flap center while maintaining approximately 5-mm thickness Adipose tissue was peeled from buccal side to slightly exceeding skin flap center while marinating approximately 5-mm thickness.

**Fig. 4:**
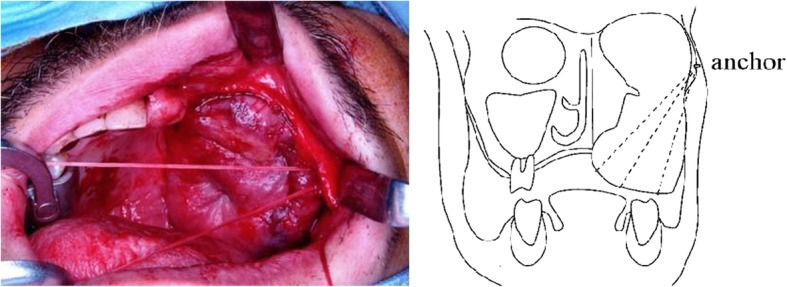
Three Mitek anchors were placed on zygomatic bone, and anchor suture was placed through subcutaneous tissue to skin flap for lifting. Skin incision was made directly above zygomatic bone with tissue separation, avoiding plate exposure, to allow easy visibility of zygomatic bone. Subcutaneous tissue was then peeled from zygomatic bone to oral cavity for tunneling. Three Mitek anchors were placed in zygomatic bone, and anchor sutures were threaded through subcutaneous tissue to lift flap

Surgical reconstruction was performed as follows:

1. An incision was made from the buccal side of the sutured edge (scar) in the abdominal rectus muscle flap (Fig. 2). We can conduct a vestibular extension at the same time by incising this position.
2. The adipose tissue was peeled from the buccal side to slightly beyond the skin flap center while maintaining approximately 5 mm thickness. The adipose tissue was reduced using a radio knife (8 g) (Fig. 3). When we reduce fat tissue, we must avoid perforating of the skin.
3. The skin was incised directly above the zygomatic bone, with tissue separation (avoiding exposure of the plate) to enable easy visibility of the zygomatic bone. Subsequently, the subcutaneous tissue was peeled from the zygomatic bone to the oral cavity for tunneling.
4. Three mini QUICKANCHOR® (Depuy Mitek Surgical Products, Inc. Raynham, MA, USA) anchors were placed in the zygomatic bone, and anchor sutures were drawn through the subcutaneous tissue to lift the skin flap. A modeling compound was used to shape the margin of the celluloid splint (Fig. 4). The advantage of flap suspension using Mitek anchors is the simple operability, less anchor positioning limitation, and easier length adjustment of the thread for suspension, which lead to easier fixation of soft tissue without slackness as well as clinically sufficient strength for fixation of ligament and tissue. On the other hand, less than 4 mm thickness of the cortical bone for suture anchor fixation causes insufficient fixation, therefore, determining placing position on the bone for fixation is necessary. Consequently, due to the versatility, the position that is considered optimal for stronger fixation and more efficient suspension can be selected as the anchor placing position, while the periosteum, corium, and scar tissue that are thought the most suitable for maintaining the strength can be chosen for the suture thread. Regarding the anchor placing position in this case, we determined 3 positions on the zygomatic bone and sutured flap corium taking into consideration a complete maxillectomy had been completed, which resulted in being able to lift the flap outward and upward.

**Fig. 5:**
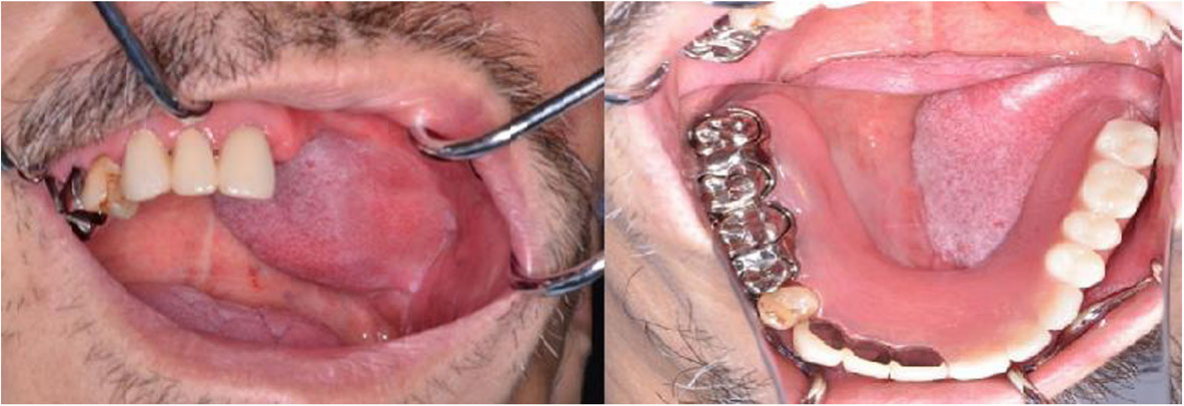
No recurrence of skin flap sagging or wound infection was observed 3 years after surgery Denture that was stabilized to right residual teeth with a clasp . Patient was quite satisfied to be able to masticate form an alimentary bolus and swallow without any teeth falling out

Postoperatively, the color of the skin flap was normal without congestion or necrosis. The celluloid splint was removed 10 days after the surgery with no infection or necrosis observed in the skin flap. We can find only fat, scar tissue, not carcinoma in the reduced fat tissure. At 3 months postoperatively, epithelialization and scarring were observed on the border of the skin flap and buccal mucosa, with no wound opening. Next, a denture that was stabilized to the right residual teeth with a clasp made. This prosthesis had two double Akers cast clasps unilaterally to retain the prosthesis by the four remaining molars. The major connector used anteria paratal plate. The patient was quite satisfied to be able to masticate, form an alimentary bolus, and swallow without any teeth falling out. No re-sagging of the skin flap or wound infection was observed at 3 years postoperatively. Patient follow-up will be continued at our department (Fig. 5).

## Discussion and conclusions

Maxillectomy can result in severe functional problems resulting from impaired mastication and deglutition [1–4]. Maxillary defects following flap reconstruction are repaired using obturator prostheses, dentures, or implants [12–14]. There are no clear selection criteria for surgical reconstruction and maxillofacial prosthetic treatments [5, 10]. Dentures can be attached immediately after surgery but do not prevent rhinolalia aperta or leakage of saliva and food [2, 12]. Implants further improve the patient’s quality of life, with superior occlusion and aesthetics as compared to dentures. Bone transplantation is often required for implants in patients with maxillary defects; however, such reconstructive surgery is not always possible. However, the osseous microvascular flap was not used, the bone which was necessary for implant was not offered. Also, the use of implant for maxillary reconstruction is controversial from a recurrence and metastatic examination and a problem such as osteoradionecrosis [15].

In cases requiring extensive excision, as with extended complete maxillectomy, covering by skin flap is sometimes essential due to exposure of the anterior cranial base and maxillary artery [14] . However, morphological reconstruction is difficult and results in frequent impairment of the denture base support and retention due to the narrow tongue space.

Although it is necessary to fully utilize the undercut in producing the prosthesis and ascertain denture retention in such cases, a secondary surgery for reshaping is required because flap sagging frequently results in a lack of denture support. Secondary modification surgeries include flap debulking, flap suspension, and alveoplasty. These methods are chosen after evaluating dental status, oroantral/nasal communication, and ablation range [4] . The right incisal tooth, canine, bicuspid, and molar were preserved in the present case and provided sufficient structure for stabilizing the artificial dentures. In such cases, implanting an anchor screw into the bone results in easier flap suspension [7–9, 11] .

The advantages of flap suspension using anchors include simplicity, fewer limitations in positioning, and easier adjustment of thread length for suspension, allowing for easier soft tissue fixation without slackening as well as clinically sufficient strength for fixation of ligaments to tissue [7–9, 11] . This device can be used in the mid-face, even if the anchor is exposed inside the maxillary sinus, enabling anchor placement at any position on the maxilla or zygomatic bone; this versatility allows for optimal anchor positioning to achieve stronger fixation and more efficient suspension. In the present case, 3 positions for anchor placement on the zygomatic bone were chosen and sutured the flap corium, taking into consideration that a complete maxillectomy had been achieved, which made it possible to lift the flap outward and upward.

The lack of relapse can be attributed to distribution of the denture weight onto the 3 anchors and suture threads and the threads through the outer layer of the corium leading to greater tensile strength. This case postoperatively presented no flap necrosis, no infection, and no reopened wound together with favorable progress, which resulted in denture stability with the satisfactory functions. Although periodical follow-up is still necessary including the denture adjustment for the future, keeping the patient informed and motivated for the periodical follow-up would be a major priority. This is because there is no sensation in the flap, which would cause difficulty in feeling the subjective symptoms such as pain, leading to a possible delay in the detection of decubital ulcer.

After removing the maxillary carcinoma, secondary reconstruction and the revision surgery imply greater complexities because it depends on case-by-case scenarios and factors such as defect conditions of osseous and soft tissue, flap conditions, degree of scar contractures, influences of preceding radiochemotherapy, the wishes of patients, and the degree of their adaptation to the patient’s social life. Also, patients’ expectations tend to be higher when undergoing a secondary operation regarding esthetic and functional improvement. In other words, their excessive expectations often result in some trouble, so that it is of great importance to conduct as many examinations as possible, such as the maximum degree of mouth opening and the masticatory ability as an objective index prior to the operation, and to give the patient sufficient informed consent together with the explanation about to what extent the function can be improved. However, the patient’s quality of life can be remarkably ameliorated when the patient understands the contents enough. Positive introduction of revision surgery is therefore necessary regarding the cases where the recurrence and metastasis are under control.

## Data Availability

All data generated or analyzed during this study are included in this published article. The datasets used and/or analyzed during the current study are available from the corresponding author on reasonable request.
